# Using a measurement type-independent metric to compare patterns of determinants between patient-reported versus performance-based physical function in hemodialysis patients

**DOI:** 10.1007/s11136-024-03745-6

**Published:** 2024-08-05

**Authors:** Gregor Liegl, Felix H. Fischer, Bernard Canaud, Mark Woodward, Claudia Barth, Andrew Davenport, Marietta Török, Giovanni F. M. Strippoli, Jörgen Hegbrant, Krister Cromm, Michiel L. Bots, Peter J. Blankestijn, Kathrin I. Fischer, Matthias Rose, the CONVINCE Scientific Committee

**Affiliations:** 1 Center for Patient-Centered Outcomes Research (CPCOR), Department of Psychosomatic Medicine, Charité—Universitätsmedizin Berlin, corporate member of Freie Universität Berlin and Humboldt-Universität Zu Berlin, 10117 Berlin, Germany; 2 School of Public Health, The George Institute for Global Health, Imperial College London, London, UK; 3 The George Institute for Global Health, University of New South Wales, Sydney, Australia; 4 Diaverum, Malmö, Sweden; 5 Department of Precision and Regenerative Medicine and Ionian Area (DiMePRe-J) University of Bari, Italy & School of Public Health, University of Sydney, Darlington, Australia; 6 Division of Nephrology, Department of Clinical Sciences, Lund University, Lund, Sweden; 7 Department of Renal Medicine, UCL, Royal Free Hospital & University College London, London, UK; 8 Global Medical Office, Fresenius Medical Care Deutschland GmbH, Bad Homburg, Germany; 9 School of Medicine, Montpellier University, Montpellier, France; 10 Julius Center for Health Sciences and Primary Care, University Medical Center Utrecht, Utrecht University, Utrecht, The Netherlands; 11 Department of Nephrology & Hypertension, University Medical Center Utrecht, Utrecht, The Netherlands; 12 Medical Scientific Affairs, B. Braun Avitum AG, Melsungen, Germany

**Keywords:** Physical function, Patient-reported outcomes, Performance outcomes, Assessment, Common metric, Hemodialysis

## Abstract

**Purpose:**

We applied a previously established common T-score metric for patient-reported and performance-based physical function (PF), offering the unique opportunity to directly compare measurement type-specific patterns of associations with potential laboratory-based, psychosocial, sociodemographic, and health-related determinants in hemodialysis patients.

**Methods:**

We analyzed baseline data from the CONVINCE trial (N = 1,360), a multinational randomized controlled trial comparing high-flux hemodialysis with high-dose hemodiafiltration. To explore the associations of potential determinants with performance-based versus patient-reported PF, we conducted multiple linear regression (backward elimination with cross-validation and Lasso regression). We used standardized T-scores as estimated from the PROMIS PF short-form 4a (patient-reported PF) and the Physical Performance Test (performance-based PF) as dependent variables.

**Results:**

Performance-based and patient-reported PF were both significantly associated with a laboratory marker-based indicator of muscle mass (simplified creatinine index), although the effects were relatively small (partial f^2^ = 0.04). Age was negatively associated with PF; the effect size was larger for performance-based (partial f^2^ = 0.12) than for patient-reported PF (partial f^2^ = 0.08). Compared to performance-based PF, patient-reported PF showed a stronger association with self-reported health domains, particularly pain interference and fatigue. When using the individual difference between patient-reported and performance-based T-scores as outcome, we found that younger age and more fatigue were associated with lower patient-reported PF compared to performance-based PF (small effect size).

**Conclusion:**

Patient-reported and performance-based assessments were similarly associated with an objective marker of physical impairment in hemodialysis patients. Age and fatigue may result in discrepancies when comparing performance-based and patient-reported scores on the common PF scale.

*Trial Registration* CONVINCE is registered in the Dutch Trial Register (Register ID: NL64750.041.18). The registration can be accessed at: https://onderzoekmetmensen.nl/en/trial/52958.

**Supplementary Information:**

The online version contains supplementary material available at 10.1007/s11136-024-03745-6.

## Introduction

Physical function is a key outcome in various medical fields [1–4], including nephrology [5–7]. According to the Patient-Reported Outcomes Measurement Information System (PROMIS) Initiative, physical function is broadly defined as the ability to perform activities that require physical actions [8–10]. This includes activities of daily living, such as self-care, but also more complex activities that require a combination of skills, often in a social context [10]. This definition covers several domains of the International Classification of Functioning, Health and Disability (ICF) by the World Health Organization [11].

Numerous types of physical function assessments exist, including highly objective measures of physiological impairment (e.g., oxygen uptake) or physical activity monitors, such as accelerometers [12]. However, when it comes to clinical outcome assessments (COAs), there is often a need for instruments that capture a broader range of everyday tasks and activities, making them more meaningful to patients’ lives [13]. For this, typically, one of two different COA types is used: patient-reported outcomes (PROs) and performance-based outcomes (PerfOs) [14, 15]. PROs and PerfOs have distinct advantages and limitations, necessitating careful consideration when choosing the appropriate instrument [13, 15]. For a valid assessment of physical function, it is advisable to consider both measurement types, as they provide different, yet complementary information [16]. To date, there is no standardized approach for meaningfully comparing PRO and PerfO results, alongside uncertainty regarding the factors necessitating consideration to allow for reliable comparisons between these modalities [15, 17].

In PROs, participants rate their perceived physical capacity by responding to standardized items, i.e., questions or statements with predefined response options [13]. PRO measures are resource-saving tools, allowing to capture a wide range of physical activities relevant to participants’ daily lives. Consequently, they show high content validity, i.e., all relevant aspects of the construct can be addressed, even when assessing a broadly defined and comprehensive physical function construct. However, due to their subjective nature, PROs are associated with symptoms like pain or depressive mood, and other patient-related factors [15, 18–20]. For example, a study found that psychosocial factors were correlated with self-reports, but not with performance-based physical function [21]. Additionally, health- and treatment-related factors may impair recall accuracy or lead to a response shift, i.e., a change in the meaning of one’s self-evaluation [22]. In this context, it has been discussed that PROs may be influenced by the specific frame of reference of the respondent, potentially introducing bias [23, 24].

As compared to PROs, PerfO measures are often discussed to be more objective but also more resource-intensive [14, 27]. That is, the functioning level is measured based on physical tasks that participants perform in a standardized setting following standardized instructions. This process demands considerable resources in terms of personnel, time, materials, and location. PerfOs are often applied as ‘single tasks’, meaning that only a specific aspect of physical function can be measured [28]. This poses a challenge in terms of content validity, as it restricts the assessment to specific subdomains of physical function [29]. However, some PerfO test batteries are available which align more closely with a broad and generic definition of physical function [15, 30]. Some recent studies indicated that such multi-task PerfO measures might have the potential to assess the same underlying construct as generic PRO measures [15, 27, 31].

In particular, the Physical Performance Test (PPT) is a frequently utilized example of comprehensive PerfO test batteries. Its items cover several subdomains, including mobility, upper extremity and fine motor skills as well as more complex activities of daily living [32]. Using item-response theory (IRT) modelling, we recently calibrated the PPT to the standardized PROMIS physical function (PROMIS PF) metric, offering the unique opportunity to directly compare patient-reported and performance-based physical function levels on the same scale [27]. For this purpose, we estimated a graded response model with fixed parameter calibration [33], whereby the parameters of the administered PROMIS PF items were fixed at their originally established values [10], while the parameters of the PPT tasks were freely estimated. The initial validation results of the established linking algorithms are promising with regard to facilitating reliable comparisons between patient-reported and performance-based physical function on the group level [27]. However, on the individual level, scores differed considerably in some cases, which is consistent with other studies indicating only moderate agreement between generic PRO and multi-task PerfO scores [34, 35].

While some factors that may contribute to disagreement between patient-reported and performance-based physical function have been discussed, evidence to date is incomplete and inconsistent, and the magnitudes of the respective effects are unclear [15, 18, 19, 25, 36]. In particular, studies comparing the differential association of PRO and PerfO scores with objective (e.g., laboratory marker-based) determinants of physical impairment such as muscle mass are missing.

Hence, this study aimed to explore whether patient-reported versus performance-based physical function scores are determined differently by various patient-level factors in hemodialysis patients. For this, we estimated PROMIS Physical Function T-scores from a PerfO and a PRO measure, using a recently established common IRT model estimated in the same study population [27]. While focusing on an objective indicator of muscle mass, we also considered additional potential laboratory, psychosocial, sociodemographic, as well as treatment- and health-related determinants of functioning.

## Materials and methods

### The CONVINCE trial

The CONVINCE trial is the largest randomized controlled trial comparing high-flux hemodialysis (HD) with high-dose hemodiafiltration (HDF) [37, 38]. Participants in dialysis centers from eight European countries (Eastern Europe: Hungary, Romania; Western Europe: France, Germany, the Netherlands, United Kingdom; and Southern Europe: Portugal, Spain), which were ≥ 18 years, diagnosed with end-stage kidney disease, and on hemodialysis for ≥ 3 month, were included, resulting in a total sample size of 1360. Recruitment took place between November 2018 and April 2021, with eligible patients randomized to receive either HDF or HD. The final follow-up assessment occurred three years after randomization. The study was conducted in full conformance with the principles of the Declaration of Helsinki. The primary outcome of the CONVINCE trial was all-cause mortality. Secondary outcomes included health-related quality of life domains, including patient-reported physical function [37]. In addition, performance-based physical function levels using the PPT were assessed at baseline. Further information on the CONVINCE trial can be found in a detailed study protocol [37] and in the publication of the main results [38].

### Participants, sample size, and handling of missing data

For the present study, we analysed data from all 1360 patients included in the CONVINCE baseline assessment. A flow diagram illustrating the sampling process up to the baseline assessment can be found in online Appendix A1. To address missing values, we employed missing value imputation using the R package ‘mice’ [39]. Predictive mean matching was used for numeric data, logistic regression imputation for binary data, polytomous regression imputation for categorical data, and a proportional odds model for ordinal data [40]. As a sensitivity analysis, we compared the resulting sample characteristics with those obtained from using another imputation method, namely classification and regression trees (CART) [40], and those obtained from a complete case analysis (see online Appendix Table A2). A detailed description of the imputation procedure is provided in online Appendix A3.

In a post-hoc power analysis, using the R package ‘pwr’ [41], a sample size of ≥ 1240 was calculated to be sufficient for detecting even very small effects (f^2^ = 0.02) in linear regression models with as many as 30 predictors (power = 0.8, significance level = 0.05).

### Measures

#### The PROMIS PF metric

The items of the PROMIS PF item bank are rated by patients themselves on a 5-point response scale, ranging from “Without any difficulty” (5) to “Unable to do” (1) or “Not at all [limited]” (5) to “Cannot do” (1), with higher-scored responses indicating a higher functioning level [42]. PROMIS PF addresses four subdomains: mobility (lower extremity), central regions (back/neck), upper extremity, and instrumental activities of daily living [10]. IRT calibration of the item bank using a graded response model (GRM) [43] allows any item subset to be used to assess an individual’s functioning level on a standardized T-score metric with a general population mean of 50 and a standard deviation (SD) of 10. Higher T-scores indicate better functioning. The PROMIS PF item bank and several short forms have been translated and validated for use in different languages [44–46]. An advantage of IRT-calibrated item banks is that they can be extended by adding new items without changing the original metric. By doing so, other physical function measures have been calibrated to the PROMIS PF T-score metric, including the performance-based PPT, allowing for comparisons of scores across these instruments [27, 47, 48].

#### The physical performance test

The PPT is a generic multi-item PerfO measure consisting of the following tasks: (1) writing a sentence, (2) simulated eating, (3) lifting a book and putting it on a shelf, (4) putting on and removing a jacket, (5) picking up a coin from the floor, (6) turning 360 degrees, (7) 50-foot walk test, (8) climbing one and (9) climbing multiple flight(s) of stairs [32]. The performance on each task is assessed on a 5-point scale by an observer, with higher scores indicating better functioning. Both the translation of the PPT and its administration followed a highly standardized approach to enable valid measurements across all participating study sites. To ensure valid measurements across countries and sites, all administrators were equipped with standardized PPT test kits and received uniform training. The PPT has been calibrated to the PROMIS T-Score metric [27].

#### The PROMIS-29 v2.0 profile

In the CONVINCE trial, patient-reported physical function was assessed with the PROMIS PF 4-item short form (PROMIS-PF4a) as part of the PROMIS-29 v2.0 profile measure [27, 37, 49]. The PROMIS-29 includes additional 4-item short forms addressing various PROMIS domains (pain interference, fatigue, depression, anxiety, sleep disturbance, and ability to participate in social roles), as well as a single item for pain intensity [49]. Each included PROMIS short form is placed on a domain-specific T-score metric, where higher scores indicate higher levels of the assessed domain (e.g. higher depression T-scores indicate more depressive mood). Each language version of every PROMIS short form utilized in CONVINCE has been translated according to standardized guidelines recommended by PROMIS [50].

#### The simplified creatinine index (SCI)

The SCI is a reliable laboratory marker-based indicator of muscle mass, serving here as an objective surrogate marker of physical impairment [51]. The SCI equation has been developed for estimating changes in skeletal muscle mass and muscle activity of hemodialysis patients and is easily calculated from usually captured laboratory parameters and anthropometric characteristics [51]. Higher values indicate more muscle mass. The SCI was found to be a reliable and valid measure with high predictive outcome value [51].

#### Additional measures

In the CONVINCE trial, commonly captured health- and treatment-related factors and sociodemographic variables were assessed [37, 38]. In addition, self-reported symptoms and psychosocial factors were measured applying an extended version of the Kidney Disease Quality of Life (KDQOL) symptom list (17 items) [52], a 5-item short form of the Perceived Stress Questionnaire (PSQ) [53], and a 5-item subset of the General Self-Efficacy Scale (GSE) [54]. Higher scores indicate more symptom burden, higher stress levels, and more self-efficacy, respectively.

For more detailed information on the data collection strategy and assessment instruments utilized in the CONVINCE study, please see the trial protocol publication [37].

### Statistical analysis

To determine the unadjusted association between muscle mass (SCI) and the different measures of physical function (PPT and PROMIS-PF4a), we calculated Spearman and Pearson correlation coefficients. We considered correlations of 0.1, 0.3, and 0.5 as small, moderate, and large, respectively [55]. To explore and compare the strength of association of the SCI and further potential determinants with performance-based versus patient-reported physical function, we conducted separate multiple linear regression models for three different dependent variables:

1. PROMIS PF T-scores obtained from the PPT (performance-based physical function)
2. PROMIS PF T-scores obtained from the PROMIS-PF4a (patient-reported physical function)
3. Delta: Difference between PROMIS T-scores obtained from the PROMIS-PF4a and the PPT for each individual patient, representing the discrepancy between patient-reported and performance-based scores. Higher delta values indicate greater discrepancy, with positive values indicating higher patient-reported scores compared to performance-based scores.

The initial models included potential determinants of physical function selected based on their availability in the CONVINCE baseline data and their assessment by clinical experts within the CONVINCE consortium as potentially relevant to physical function:

#### Measures of body composition

- SCI (mg/kg/day)
- Body Mass Index (BMI: kg/m.^2^)

#### Laboratory markers

- Hemoglobin g/dl
- Calcium mg/dl
- Phosphate mg/dl
- Sodium mmol/l
- Creatinine mg/dl
- Dialysis adequacy (Kt/V)

#### Health- and treatment-related variables

- Co-morbid conditions (diabetes, cancer, cardiovascular disease, chronic pulmonary obstructive disease)
- Dialysis vintage
- Medication (erythropoiesis stimulating agents, iron preparations)

#### Socio-demographic variables

- Age
- Sex
- Educational status: primary (reference category), lower secondary, upper secondary, tertiary
- Region: Western Europe (reference category), Eastern Europe, Southern Europe

#### Patient-reported factors

- Depression
- Fatigue
- Anxiety
- Sleep disturbance
- Pain interference
- Symptom burden
- Self-efficacy
- Perceived stress

Two PROMIS-29 domains were not included as independent variables. First, *ability to participate in social roles and activities* was considered to be conceptually too close to the above-mentioned generic definition of physical function. Second, *pain intensity* was excluded because of very high correlations with the *pain interference* domain.

Given that the SCI is calculated based on other variables included in the above list of determinants, we estimated two separate models for each dependent variable with either the SCI (Model 1) or the individual components used to calculate the SCI (Model 2) included. Stepwise backward regression with tenfold cross-validation was applied to refine each model [56]. We utilized the Akaike Information Criterion (AIC) for the backward elimination procedure, iteratively removing independent variables until achieving the model with the best fit. Additionally, to ensure the robustness of our findings, we conducted a sensitivity analysis by comparing the results with those obtained from Lasso regression, a regularized regression approach particularly suitable for models with a large number of predictors [57]. Lasso regression helps address issues of multicollinearity and overfitting by penalizing the absolute size of the regression coefficients, promoting sparsity in the model.

Effect sizes for determinants were assessed using the f^2^ statistic. We considered f^2^ values of 0.02, 0.15, and 0.35 as indicative of small, medium, and large effects, respectively [55].

Before interpreting the results of the refined models using backward elimination of determinants, we verified the following assumptions of linear regression analysis:

- 1. Cook’s distance values were calculated to detect significant outliers (cut-off of ≥ 1).
- 2. Variance inflation factors were computed to verify the absence of multicollinearity among independent variables (cut-off of > 10).
- 3. Homoscedasticity of residuals was investigated using the Goldfeld-Quandt test (α = 0.01).
- 4. We graphically assessed the assumptions of linearity between dependent and independent variables and of normality of residuals.

We used R version 4.2.1 for statistical analyses [58]. For regression analyses, we applied the packages ‘car’ [59], ‘caret’ [60], ‘glmnet’ [61], and ‘MASS’ [62].

## Results

The sample characteristics, presented in Table 1, demonstrated a high degree of similarity in sociodemographic and clinical features before and after missing value imputation. Additionally, the sensitivity analysis, detailed in online Appendix Table A2, suggested a negligible impact resulting from the specific method employed for data imputation.

**Table 1:** Summary statistics of sample characteristics (N = 1360)

	Sample with missing data	Sample with imputed data	*Missing / imputed values; n (%)*
Female; n (%)	504 (37.1)	504 (37.1)	*0 (0.0)*
Age in years; mean (SD)	62.4 (13.5)	62.4 (13.5)	*0 (0.0)*
BMI; mean (SD)	27.4 (5.6)	27.4 (5.6)	*9 (0.7)*
SCI (mg/kg/day); mean (SD)	19.7 (2.5)	19.7 (2.4)	*208 (15.3)*
Hemoglobin g/dl; mean (SD)	11.3 (1.2)	11.3 (1.2)	*15 (1.1)*
Calcium mg/dl; mean (SD)	8.9 (0.7)	8.9 (0.7)	*67 (4.9)*
Phosphate mg/dl; mean (SD)	4.9 (1.5)	4.9 (1.5)	*36 (2.6)*
Sodium mmol/l; mean (SD)	137.9 (3.2)	137.9 (3.2)	*26 (1.9)*
Serum creatinine mg/dl; mean (SD)	8.3 (2.4)	8.3 (2.4)	*158 (11.6)*
Kt/V; mean (SD)	1.7 (0.5)	1.7 (0.5)	*69 (5.1)*
Dialysis vintage in months; median (IQR)	33 (56)	33 (56)	*3 (0.2)*
Medication; n (%)			*0 (0.0)*
ESA	1009 (74.2)	1009 (74.2)	
Iron preparations	862 (63.4)	862 (63.4)	
Co-morbid conditions; n (%)			*1 (0.1)*
Diabetes	481 (35.4)	481 (35.4)	
CVD	598 (44.0)	598 (44.0)	
Cancer	181 (13.3)	181 (13.3)	
COPD	101 (7.4)	101 (7.4)	
Education			*136 (10.0)*
Less than secondary	202 (16.5)	249 (18.3)	
Lower secondary	438 (35.8)	463 (34.0)	
Upper secondary	400 (32.7)	446 (32.8)	
Tertiary (Bachelor or higher)	184 (15.0)	202 (14.9)	
Region; n (%)			*0 (0.0)*
Eastern European	467 (34.3)	467 (34.3)	
Western European	441 (32.4)	441 (32.4)	
Southern European	452 (33.2)	452 (33.2)	
Physical function			
Patient-reported (PRO)*; mean (SD)	42.8 (8.9)	42.7 (8.9)	*102 (7.5)*
Performance (PerfO)*; mean (SD)	42.6 (9.5)	42.5 (9.6)	*167 (12.3)*
Delta (PRO—PerfO); mean (SD)	0.1 (8.5)	0.2 (8.6)	*228 (16.7)*
Depression*; mean (SD)	50.3 (9.0)	50.4 (9.0)	*93 (6.8)*
Fatigue*; mean (SD)	50.3 (9.3)	50.3 (9.3)	*78 (5.7)*
Anxiety*; mean (SD)	49.4 (9.3)	49.3 (9.3)	*82 (6.0)*
Sleep disturbance*; mean (SD)	49.0 (9.3)	48.9 (9.3)	*78 (5.7)*
Pain interference*; mean (SD)	51.9 (9.7)	52.0 (9.7)	*78 (5.7)*
Symptom burden^§^; mean (SD)	28.9 (9.4)	28.9 (9.3)	*153 (11.3)*
Self-efficacy^‡^; mean (SD)	15.8 (3.5)	15.8 (3.5)	*100 (7.4)*
Perceived stress^‡^; mean (SD)	13.4 (3.6)	13.4 (3.5)	*92 (6.8)*

BMI, body mass index; COPD, chronic obstructive pulmonary disease; CVD, cardiovascular disease; IQR, interquartile range; SCI, simplified creatinine index; SD, standard deviation

^*^T-scores as measured on the corresponding PROMIS domain; higher scores indicate higher levels of the underlying construct (e.g., more pain interference)

^†^As measured by a single item with a 0 to 10 rating scale; higher scores indicate more severe pain

^§^Sum score of a 17-items symptom list (5-response options per item) with a minimum of 17 and a maximum of 85; higher scores indicate more symptom burden

^‡^Sum score of 5-item short forms (5-response options per item) with a minimum of five and a maximum of 20; higher scores indicate more of the assessed construct

**Table 2:** Regression model with performance-based physical function as dependent variable (outcome) after stepwise elimination of indicators

Outcome	Determinants	Model 1 (including SCI)				Model 2 (with individual SCI components)			
		R^2^ / adj. R^2^	Beta	95% CI	partial f^2^	R^2^ / adj. R^2^	Beta	95% CI	partial f^2^
PPT	Model	26.1 / 25.1				32.5 / 31.4			
	SCI		0.73	0.53; 0.92*	0.040		*n.a*	*n.a*	*n.a*
	Age		*n.a*	*n.a*	*n.a*		− 0.24	− 0.27; − 0.20*	0.124
	BMI		− 0.21	− 0.29; − 0.11*	0.018		− 0.20	− 0.29; − 0.12*	0.018
	Educational level^†^								
	Lower secondary		2.16	0.83; 3.49*	0.008		1.61	0.41; 2.91*	0.005
	Upper secondary		4.35	2.98; 5.67*	0.027		2.63	2.35; 4.90*	0.021
	Tertiary		2.81	1.09; 4.44*	0.009		2.26	0.65; 3.85*	0.006
	Laboratory measures								
	Hemoglobin		0.28	− 0.11; 0.68	0.002		0.36	− 0.03; 0.75	0.003
	Sodium		0.20	0.06; 0.35*	0.006		0.23	0.10; 0.36*	0.008
	Phosphate		*n.a*	*n.a*	*n.a*		− 2.22	− 0.55; 0.11	0.001
	Kt/V		*n.a*	*n.a*	*n.a*		− 1.47	− 2.53; − 0.51*	0.008
	Comorbidities								
	Diabetes		− 2.32	− 3.43; − 1.36*	0.015		− 2.13	− 3.04; − 1.13*	0.014
	Cancer		*n.a*	*n.a*	*n.a*		1.70	0.49; 2.88*	0.005
	CVD		− 2.84	− 3.76; − 1.85*	0.026		− 1.72	− 2.70; − 0.82*	0.010
	COPD		− 2.27	− 4.11; − 0.54*	0.005		− 1.44	− 3.15; 0.42	0.002
	Treatment-related factors								
	Iron preparations		2.61	1.61; 3.53*	0.022		2.50	1.53; 3.39*	0.022
	Patient-reported variables								
	Depression		− 0.08	− 0.17; 0.00	0.003		− 0.11	− 0.20; − 0.03*	0.006
	Fatigue		− 0.07	− 0.14; 0.00	0.003		− 0.06	− 0.13; 0.00	0.003
	Anxiety		0.07	0.00; 0.15	0.002		0.06	− 0.01; 0.14	0.002
	Pain Interference		− 0.13	− 0.19; − 0.06*	0.013		− 0.12	− 0.18; − 0.06*	0.012
	Self-efficacy		0.15	0.00; 0.29	0.003		0.12	− 0.02; 0.26	0.002
	Region^§^								
	Eastern European		− 2.22	− 3.35; − 0.95*	0.011		− 2.84	− 4.02; − 1.72*	0.018
	Southern European		− 1.50	− 2.60; − 0.35*	0.005		− 1.92	− 3.02; − 0.87*	0.008

BMI, body mass index; CVD, cardiovascular disease; CI, confidence interval; COPD, chronic obstructive pulmonary disease;

Kt/V, a measure of hemodialysis adequacy; PPT, Physical Performance Test; SCI, simplified creatinine index

^†^Reference category: “Less than secondary”

^§^Reference category: “Western European”

^*^95% CI indicates statistical significance

**Fig. 1:**
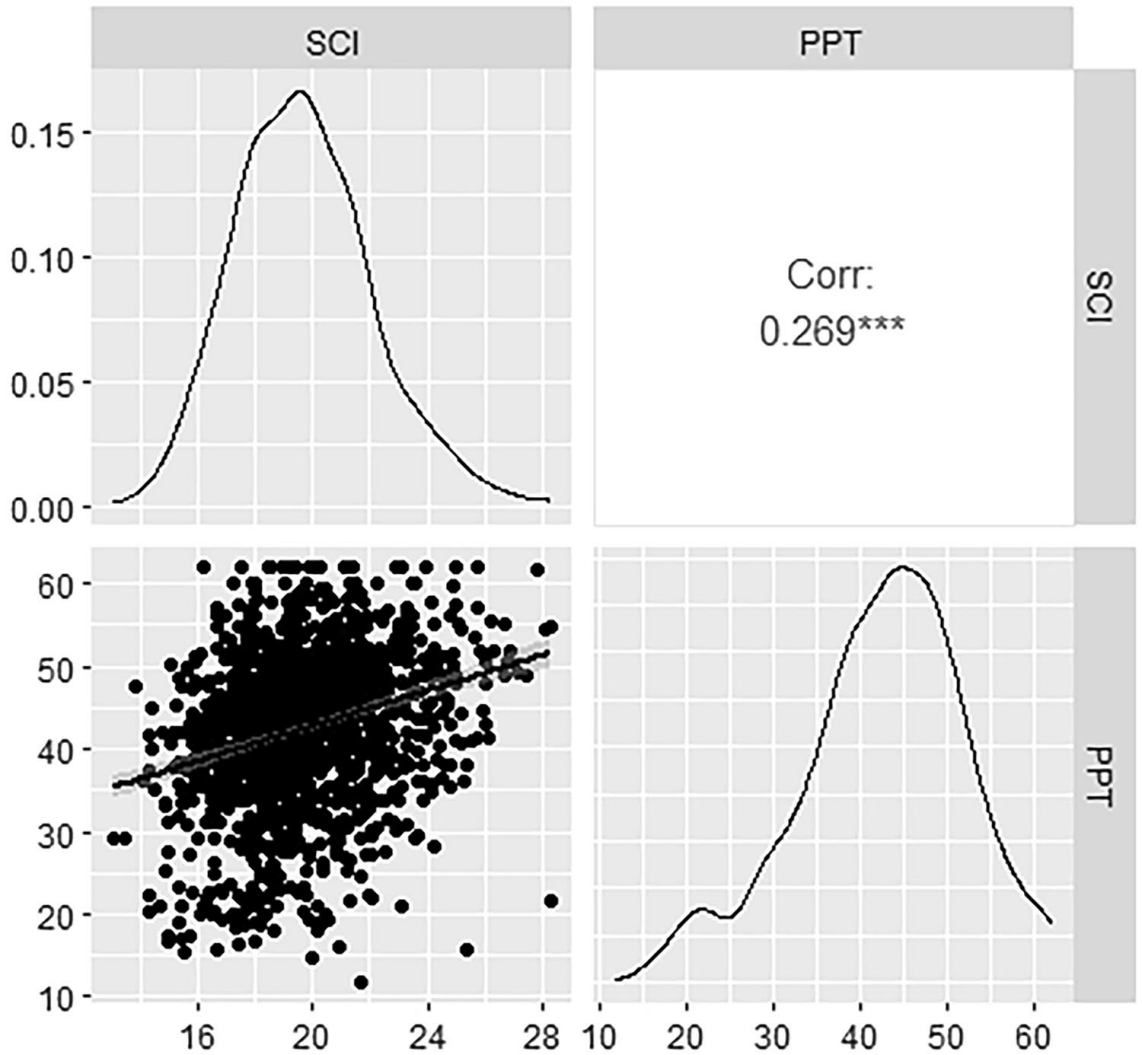
Pearson correlation coefficient (top right box) and scatter plot (bottom left box) for the relationship between SCI and performance-based physical function as assessed with the PPT, and density plots visualizing the distributions of each measure (diagonal boxes). ***Correlation is statistically significant (P < .001). Corr, correlation; PPT, Physical Performance Test

**Fig. 2:**
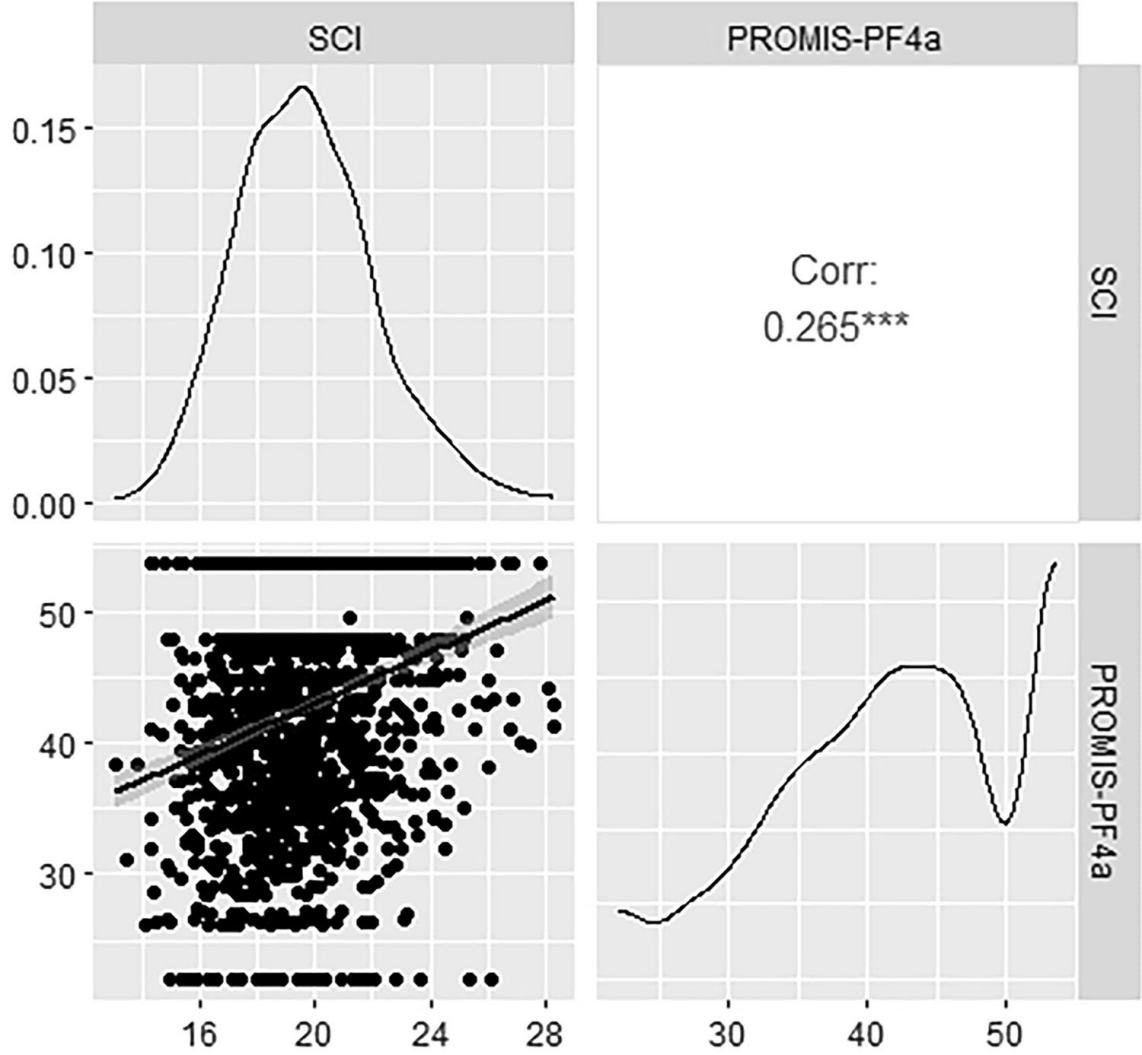
Pearson correlation coefficient (top right box) and scatter plot (bottom left box) for the relationship between SCI and patient-reported physical function as assessed with the PROMIS-PF4a, and density plots visualizing the distributions of each measure (diagonal boxes). ***Correlation is statistically significant (P < .001). Corr, correlation; PF, physical function; PROMIS-PF4a, Patient-Reported Outcomes

We found moderate but statistically significant Pearson correlations between the SCI and physical function scores, as depicted in Figs. 1 and 2 along with score distributions and scatter plots. Spearman correlation coefficients demonstrated similar relationships, with r = 0.272 for SCI with performance-based and r = 0.273 with patient-reported physical function. Figure 2 indicated the presence of ceiling effects in the PROMIS-PF4a measure.

Assumptions of regression analysis were largely met. We observed slight deviations from normality of the residuals in the models with the PPT and PROMIS-PF4a as outcomes. Thus, to provide 95% confidence intervals of the regression coefficients, we employed bootstrapping with 2000 samples.

Table 2 summarizes the results of the backward regression with cross-validation for both models with performance-based physical function as dependent variable. While several indicators were selected after stepwise elimination of independent factors, most effect sizes were negligible or very small (partial f^2^ close to 0.02). We found small-to-moderate effect sizes for the SCI (partial f^2^ = 0.04) and age (partial f^2^ = 0.12).

Results of the stepwise regression models with patient-reported physical function as dependent variable after stepwise elimination of indicators are presented in Table 3. Again, we found small-to-moderate effect sizes for the SCI (partial f^2^ = 0.04) and age (partial f^2^ = 0.08); however, age was associated less with patient-reported than with performance-based physical function. This difference was not observed for the SCI, as indicated by non-overlapping confidence intervals. Moreover, we found small-to-moderate effect sizes regarding negative associations of patient-reported physical function with fatigue (partial f^2^ = 0.06) and pain interference (partial f^2^ = 0.06).

**Table 3:** Regression model with patient-reported physical function as dependent variable (outcome) after stepwise elimination of indicators

Outcome	Determinants	Model 1 (including SCI)				Model 2 (with individual SCI components)			
		R^2^ / adj. R^2^	Beta	95% CI	partial f^2^	R^2^ / adj. R^2^	Beta	95% CI	partial f^2^
PROMIS PF-4a	Model	50.9 / 50.3				52.8 / 52.2			
	SCI		0.54	0.38; 0.69*	0.039		*n.a*	*n.a*	*n.a*
	Age		*n.a*	*n.a*	*n.a*		− 0.14	− 0.17; − 0.11*	0.075
	Sex		*n.a*	*n.a*	*n.a*		− 0.98	− 1.66; -− 0.22*	0.005
	BMI		− 1.12	− 0.18; − 0.06*	0.010		− 0.11	− 0.17; − 0.04*	0.008
	Educational level^†^								
	Lower secondary		1.20	0.11; 2.25*	0.004		0.92	− 0.12; 2.03	0.003
	Upper secondary		2.03	1.00; 3.16*	0.012		1.60	0.50; 2.68*	0.008
	Tertiary		1.49	0.31; 2.77*	0.004		1.16	− 0.04; 2.31	0.003
	Laboratory measures								
	Hemoglobin		0.38	0.09; 0.63*	0.005		0.41	0.15; 0.67*	0.007
	Sodium		0.08	− 0.02; 0.20	0.002		0.10	0.00; 0.20	0.002
	Kt/V		*n.a*	*n.a*	*n.a*		− 0.59	− 1.34; 0.09	0.002
	Comorbidities								
	Diabetes		− 1.56	− 2.40; − 0.76*	0.012		− 1.59	− 2.37; − 0.86*	0.013
	Cancer		− 0.84	− 1.78; 0.14	0.002		*n.a*	*n.a*	*n.a*
	CVD		− 1.91	− 2.65; − 1.18*	0.021		− 1.39	− 2.20; − 0.62*	0.011
	COPD		− 2.46	− 3.71; − 1.10*	0.010		− 2.21	− 3.45; − 1.06*	0.008
	Patient-reported variables								
	Depression		− 0.07	− 0.12; − 0.01*	0.005		− 0.08	− 0.14; − 0.03*	0.007
	Fatigue		− 0.24	− 0.29; − 0.18*	0.059		− 0.23	− 0.28; − 0.17*	0.058
	Pain Interference		− 0.21	− 0.26; − 0.16*	0.059		− 0.20	− 0.25; − 0.15*	0.055
	Symptom burden		− 0.12	− 0.17; − 0.07*	0.015		− 0.12	− 0.17; − 0.07*	0.016
	Perceived stress		− 0.07	− 0.18; 0.03	0.002		*n.a*	*n.a*	*n.a*
	Self-efficacy		0.18	0.06; 0.30*	0.008		0.17	0.06; 0.30*	0.008

BMI, body mass index; CVD, cardiovascular disease; CI, confidence interval; COPD, chronic obstructive pulmonary disease; Kt/V, a measure of hemodialysis adequacy; *n.a.*, not applicable; PPT, Physical Performance Test; PROMIS-PF4a, Patient-Reported Outcomes Measurement Information System Physical Function 4a; SCI, simplified creatinine index

^†^Reference category: “Less than secondary”

^*^95% CI indicates statistical significance

Regression results of the models with the T-score difference between PROMIS-PF4a versus PPT as dependent variable after stepwise elimination of indicators are presented in Table 4. We found a small relationship between age and higher delta values (partial f^2^ = 0.03), suggesting that older patients tend to rate their physical function higher relative to their actual performance. Moreover, fatigue was negatively associated with T-score differences between the PROMIS-PF4a and the PPT (small effect size), indicating that higher fatigue was associated with lower patient-reported physical function relative to T-scores based on actual performance.

**Table 4:** Regression model with T-score differences between PROMIS-PF4a versus PPT as dependent variable after stepwise elimination of indicators

Outcome	Determinants	Model 1 (including SCI)				Model 2 (with individual SCI components)			
		R^2^ / adj. R^2^	Beta	95% CI	partial f^2^	R^2^ / adj. R^2^	Beta	95% CI	partial f^2^
Delta (PROMIS PF4a—PPT)	Model	18.8 / 17.8				21.0 / 20.1			
	SCI		− 0.19	− 0.40; − 0.01*	0.003		*n.a*	*n..a*	*n.a*
	Age		*n.a*	*n..a*	*n.a*		0.11	0.08; 0.14*	0.033
	Sex		*n.a*	*n..a*	*n.a*		− 1.42	− 2.34; − 0.53*	0.007
	BMI		0.08	0.00; 0.17	0.003		0.10	0.03; 0.18*	0.005
	Educational level†								
	Lower secondary		− 0.97	− 2.27; 0.24	0.002		*n.a*	*n..a*	*n.a*
	Upper secondary		− 2.37	− 3.68; − 1.00*	0.009		− 1.36	− 2.26; -0.42*	0.006
	Tertiary		− 1.34	− 2.95; 0.16	0.002		*n.a*	*n..a*	*n.a*
	Laboratory measures								
	Phosphate		*n.a*	*n..a*	*n.a*		0.24	− 0.06; 0.54	0.002
	Sodium		− 0.12	− 0.25; 0.02	0.002		− 0.15	− 0.27; − 0.03*	0.004
	Kt/V		*n.a*	*n..a*	*n.a*		0.93	0.02; 1.95*	0.003
	Comorbidities								
	Diabetes		0.78	− 0.11; 1.70	0.002		*n.a*	*n..a*	*n.a*
	Cancer		− 1.36	− 2.51; − 0.09*	0.003		− 1.98	− 3.20; − 0.80*	0.007
	CVD		0.89	0.00; 1.73	0.003		*n.a*	*n..a*	*n.a*
	Treatment-related factors								
	ESA		− 0.71	− 1.69; 0.26	0.002		*n.a*	*n..a*	*n.a*
	Iron preparations		− 2.09	− 3.15; − 1.23*	0.015		− 2.13	− 2.97; − 1.27*	0.017
	Patient-reported variables								
	Fatigue		− 0.18	− 0.25; − 0.12*	0.024		− 0.18	− 0.25; − 0.12*	0.025
	Sleep disturbance		*n.a*	*n..a*	*n.a*		− 0.04	− 0.09; 0.02	0.002
	Pain interference		− 0.09	− 0.15; − 0.04*	0.008		− 0.09	− 0.15; − 0.04*	0.008
	Anxiety		− 0.05	−0.10; 0.01	0.002		*n.a*	*n..a*	*n.a*
	Symptom burden		− 0.10	− 0.17; − 0.02*	0.007		− 0.09	− 0.17; − 0.02*	0.006
	Perceived Stress		− 0.13	− 0.25; − 0.01*	0.004		− 0.13	− 0.24; − 0.01*	0.004
	Region^§^								
	Eastern European		2.41	1.27; 3.55*	0.014		2.72	1.59; 3.72*	0.018
	Southern European		1.58	0.58; 2.51*	0.006		1.87	0.83; 2.83*	0.009

CI, confidence interval; CVD, cardiovascular disease; ESA, erythropoiesis-stimulating agent; Kt/V, a measure of hemodialysis adequacy; *n.a.*, not applicable; PPT, Physical Performance Test; PROMIS-PF4a, Patient-Reported Outcomes Measurement Information System Physical Function 4a; SCI, simplified creatinine index

^†^Reference category: “Less than secondary”

^§^Reference category: “Western European”

^*^95% CI indicates statistical significance

The sensitivity analysis comparing the results of the backward selection with those obtained from Lasso regression is detailed in online Appendix Tables A4 to A6. In general, the Lasso regression tended to retain more independent variables in the model (even with very small effect sizes). However, the coefficients of the variables retained by both models were not significantly different from each other.

## Discussion

This study aimed to directly compare patterns of individual-level determinants associated with patient-reported versus performance-based physical function assessments in patients undergoing hemodialysis, including laboratory, psychosocial, sociodemographic, as well as treatment- and health-related factors. This was made possible because, in a prior study, we used IRT modeling to link the performance-based PPT onto the well-established PROMIS PF T-score metric [27]. Our findings have important implications for understanding the determinants of physical function in this population.

Both patient-reported and performance-based assessments demonstrated similar associations with an objective measure of muscle mass, specifically the SCI. This supports the idea that both COA types can offer valuable information about physical health [15], even though PROs are more subjective. However, the association of the SCI with both COA types was relatively weak, implying that laboratory measures may not be reliable indicators of functioning. In this context, none of the other investigated laboratory measures showed any relevant associations with physical functioning, aligning with findings from previous studies in hemodialysis patients [63]. Hence, the association between physical function and the SCI may primarily stem from age, which is a relevant indicator of functioning and a component of the SCI formula [51]. The limited effectiveness of laboratory measures in assessing functional status highlights the importance of employing patient-centered assessment tools to evaluate the patient’s actual functioning and perceived health.

Another finding of our study is that, in comparison to performance assessments, patient-reported physical function was considerably more associated with other patient-reported health domains, particularly symptoms such as fatigue and pain. The association of pain with patient-reported physical functioning is well-known from the literature [15, 18, 20]. Another patient-reported factor sometimes considered a significant predictor of self-reported physical functioning is depression [19, 25]. Interestingly, in our study, when fatigue was excluded as a determinant from the regression model, depression emerged as a significant factor (data not shown). This suggests that cognitive/emotional fatigue, which is closely linked to depression [64], might be the primary explanatory factor. In summary, patient-reported constructs of physical function might represent more than just capability, but ‘capability free from symptomatic constraints’. These findings underscore the importance of considering factors such as fatigue and pain when interpreting patient-reported physical function assessments in clinical settings. However, it cannot be ruled out that the higher correlations across patient-reported variables might be partly due to a method effect, meaning that the specific assessment method (e.g., PRO) contributes variance to scores beyond the constructs of interest [65].

The study revealed that higher age had a more pronounced negative effect on performance-based compared to patient-reported physical function, which has also been indicated by the results of previous studies [15, 25, 26]. On the other hand, fatigue was associated with lower patient-reported relative to performance-based functioning. These findings suggest that age and cognitive/emotional fatigue may lead to discrepancies when directly comparing both COA types on a common scale. While the impact of these factors is expected to be small, it remains crucial for clinicians and researchers to take them into account when interpreting findings, particularly when assessing patient-reported functioning in fatigued individuals. Moreover, on the group level, statistical adjustments or subgroup analyses could be considered.

This study has limitations. First, as discussed earlier [27], the PROMIS-PF4a exhibited ceiling effects, potentially leading to somewhat biased comparisons between patient-reported and performance-based scores. Future studies using more comprehensive PRO measures allowing to apply and compare alternative methods for linking PRO and PerfO measures [66] are required to address this issue. Second, we analyzed data from a randomized controlled trial, which may not have captured all potential determinants of physical function and all potential confounders affecting the relationship between these determinants and the measured physical function outcome. Third, this study focused on hemodialysis patients and the utilized common metric was based on data from the same sample. Future research could explore a broader range of factors that might influence physical function in hemodialysis patients, but also in different patient cohorts to determine the generalizability of our findings. Fourth, missing values were present in the dataset. Despite efforts to mitigate this issue using multiple imputation techniques, missing data may have introduced some degree of bias. Fifth, this study rests on the assumption of country-, language-, and culture-related measurement invariance of the utilized patient-reported and performance measures. This assumption could not be examined for each measure and each individual country, which should be taken into account when interpreting our findings. However, with regard to the PROMIS-PF4a and the PPT, a previous analysis of CONVINCE baseline data did not indicate relevant differential item functioning across Eastern, Southern, and Western European countries [27]. All measures were translated and culturally adapted according to rigorous guidelines. In particular for various PROMIS measures, numerous studies indicating measurement invariance between different countries and language versions exist [46, 67–69].

Despite these limitations, one of the study’s significant contributions is highlighting the advantages of using a common metric for PRO and PerfO measures. Even though both COA types were not perfectly correlated, having a common metric allowed for meaningful comparisons of related instruments. This approach enhances the interpretability of results and simplifies the comparison of data from different measurement types. While both PROs are PerfOs are recommended for clinical outcome assessments [13, 16], currently, the lack of a reliable method to compare results from these assessment types poses challenges for clinical practice and healthcare policy decision-making. Establishing a common metric, coupled with the identification of instrument-specific determinants, offers a promising approach to enhance the validity and usefulness of outcome measures.

## Conclusion

This study compared patient-reported and performance-based physical function in hemodialysis patients, using a common T-score metric. Both assessment types exhibited similar associations with a laboratory-based measure of muscle mass. Compared to performance assessments, patient-reported physical function was considerably more associated with other patient-reported variables. These findings underscore the importance of comprehensive assessment strategies in clinical practice and highlight the need to consider both patient-reported and performance-based measures to gain a comprehensive understanding of physical function in individuals with chronic conditions, such as end-stage kidney disease. A limitation of this study is its reliance on data from a specific study population. Future studies should aim to replicate our findings in diverse patient populations which would enhance the generalizability of the findings.

## Supplementary Information

Below is the link to the electronic supplementary material.

Supplementary file1 (DOCX 69 KB)

Supplementary file2 (DOCX 28 KB)

Supplementary file3 (DOCX 21 KB)

Supplementary file4 (DOCX 51 KB)

## Data Availability

Not applicable.
