# Supplementary figures and images for: Using a measurement type-independent metric to compare patterns of determinants between patient-reported versus performance-based physical function in hemodialysis patients
Source: Qual Life Res. 2024 Aug 5;33(11):2987–3001. doi: 10.1007/s11136-024-03745-6 (PMC11541257; doi:10.1007/s11136-024-03745-6)

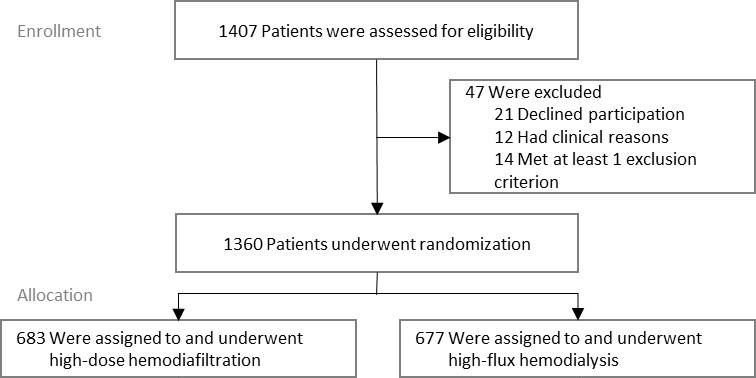

Supplement: Supplementary file 1 — Supplementary file1 (DOCX 69 KB) [file 11136_2024_3745_MOESM1_ESM.docx]
