# Supplementary material for: Using a measurement type-independent metric to compare patterns of determinants between patient-reported versus performance-based physical function in hemodialysis patients
Source: Qual Life Res. 2024 Aug 5;33(11):2987–3001. doi: 10.1007/s11136-024-03745-6 (PMC11541257; doi:10.1007/s11136-024-03745-6)
Supplement: Supplementary file 2 — Supplementary file2 (DOCX 28 KB) [file 11136_2024_3745_MOESM2_ESM.docx]

| Table A2: Summary statistics of sample characteristics - Sensitivity analysis | | | | | | |
| --- | --- | --- | --- | --- | --- | --- |
|  | | | Utilized  imputation^¶^ (n=1360) | CART imputation^¶^ (n=1360) | Complete case analysis (n=815) | *Imputed values,*  *n (%)* |
| Female; n (%) | | 504 (37.1) | | 504 (37.1) | 293 (36.0) | *0 (0.0)* |
| Age in years; mean (SD) | | 62.4 (13.5) | | 62.4 (13.5) | 61.3 (13.2) | *0 (0.0)* |
| BMI; mean (SD) | | 27.4 (5.6) | | 27.4 (5.7) | 27.6 (5.7) | *9 (0.7)* |
| SCI (mg/kg/day); mean (SD) | | 19.7 (2.4) | | 19.7 (2.4) | 19.7 (2.4) | *208 (15.3)* |
| Hemoglobin g/dl; mean (SD) | | 11.3 (1.2) | | 11.3 (1.2) | 11.2 (1.2) | *15 (1.1)* |
| Calcium mg/dl; mean (SD) | | 8.9 (0.7) | | 8.9 (0.7) | 8.8 (0.7) | *67 (4.9)* |
| Phosphate mg/dl; mean (SD) | | 4.9 (1.5) | | 4.9 (1.5) | 5.0 (1.5) | *36 (2.6)* |
| Sodium mmol/l; mean (SD) | | 137.9 (3.2) | | 137.9 (3.2) | 138.0 (3.2) | *26 (1.9)* |
| Serum creatinine mg/dl; mean (SD) | | 8.3 (2.4) | | 8.3 (2.3) | 8.3 (2.3) | *158 (11.6)* |
| Kt/V; mean (SD) | | 1.7 (0.5) | | 1.7 (0.5) | 1.7 (0.5) | *69 (5.1)* |
| Dialysis vintage in months; median (IQR) | | 33 (56) | | 33 (56) | 34 (59) | *3 (0.2)* |
| Medication; n (%) | |  | |  |  | *0 (0.0)* |
| ESA | 1009 (74.2) | | | 1009 (74.2) | 591 (72.5) |  |
| Iron preparations | 862 (63.4) | | | 862 (63.4) | 508 (62.3) |  |
| Co-morbid conditions; n (%) | |  | |  |  | *1 (0.1)* |
| Diabetes | | 481 (35.4) | | 481 (35.4) | 284 (34.8) |  |
| CVD | | 598 (44.0) | | 598 (44.0) | 362 (44.4) |  |
| Cancer | | 181 (13.3) | | 181 (13.3) | 100 (12.3) |  |
| COPD | | 101 (7.4) | | 101 (7.4) | 66 (8.1) |  |
| Education | |  | |  |  | *136 (10.0)* |
| Less than secondary | | 249 (18.3) | | 249 (18.3) | 139 (17.1) |  |
| Lower secondary | | 463 (34.0) | | 463 (34.0) | 258 (31.7) |  |
| Upper secondary | | 446 (32.8) | | 446 (32.8) | 292 (35.8) |  |
| Tertiary (Bachelor or higher) | | 202 (14.9) | | 202 (14.9) | 126 (15.4) |  |
| Region; n (%) | |  | |  |  | *0 (0.0)* |
| Eastern European | | 467 (34.3) | | 467 (34.3) | 359 (44.0) |  |
| Western European | | 441 (32.4) | | 441 (32.4) | 224 (27.5) |  |
| Southern European | | 452 (33.2) | | 452 (33.2) | 232 (28.5) |  |
| Physical function | |  | |  |  |  |
| Patient-reported (PRO)*; mean (SD) | | 42.7 (8.9) | | 42.7 (8.9) | 43.1 (8.8) | *102 (7.5)* |
| Performance (PerfO)*; mean (SD) | | 42.5 (9.6) | | 42.6 (9.4) | 43.1 (9.4) | *167 (12.3)* |
| Delta (PRO – PerfO); mean (SD) | | 0.2 (8.6) | | 0.1 (8.4) | 0.0 (8.4) | *228 (16.7)* |
| Depression*; mean (SD) | | 50.4 (9.0) | | 50.4 (9.0) | 50.4 (9.1) | *93 (6.8)* |
| Fatigue*; mean (SD) | | 50.3 (9.3) | | 50.3 (9.3) | 50.3 (9.2) | *78 (5.7)* |
| Anxiety*; mean (SD) | | 49.3 (9.3) | | 49.4 (9.3) | 49.5 (9.2) | *82 (6.0)* |
| Sleep disturbance*; mean (SD) | | 48.9 (9.3) | | 49.0 (9.3) | 48.8 (9.5) | *78 (5.7)* |
| Pain interference*; mean (SD) | | 52.0 (9.7) | | 51.9 (9.7) | 52.4 (9.7) | *78 (5.7)* |
| Symptom burden^§^; mean (SD) | | 28.9 (9.3) | | 28.9 (9.4) | 29.2 (9.3) | *153 (11.3)* |
| Self-efficacy^‡^; mean (SD) | | 15.8 (3.5) | | 15.7 (3.6) | 15.7 (3.5) | *100 (7.4)* |
| Perceived stress^‡^; mean (SD) | | 13.4 (3.5) | | 13.4 (3.5) | 13.6 (3.3) | *92 (6.8)* |

*Abbreviations:* BMI, body mass index; CART, Classification and Regression Trees, COPD, chronic obstructive pulmonary disease; CVD, cardiovascular disease; IQR, interquartile range; SCI, simplified creatinine index; SD, standard deviation.

* T-scores as measured on the corresponding PROMIS domain; higher scores indicate higher levels of the underlying construct (e.g., more pain interference).

^†^ As measured by a single item with a 0 to 10 rating scale; higher scores indicate more severe pain.

^§^ Sum score of a 17-items symptom list (5-response options per item) with a minimum of 17 and a maximum of 85; higher scores indicate more symptom burden.

^‡^ Sum score of 5-item short forms (5-response options per item) with a minimum of five and a maximum of 20; higher scores indicate more of the assessed construct.

^¶^ Values from the first of five imputed datasets
