# Supplementary material for: Using a measurement type-independent metric to compare patterns of determinants between patient-reported versus performance-based physical function in hemodialysis patients
Source: Qual Life Res. 2024 Aug 5;33(11):2987–3001. doi: 10.1007/s11136-024-03745-6 (PMC11541257; doi:10.1007/s11136-024-03745-6)
Supplement: Supplementary file 3 — Supplementary file3 (DOCX 21 KB) [file 11136_2024_3745_MOESM3_ESM.docx]

**Appendix A3: Handling of missing data**

The following section describes the handling of missing values and the data imputation procedure. We followed the reporting guidelines outlined by Van Buuren (2018) in ‘Flexible Imputation of Missing Data’, Chapter 12.2.

The percentage of missing values across all 31 variables used for analysis in this study is shown in both Table 1 and Appendix Table A2. Only three of the original variables (serum creatinine, performance-based physical function, symptom burden) and two derived variables (SCI, and the difference between performance-based and patient-reported physical function) had more than 10% missing values. Overall, data from 545 out of 1360 participants (40%) were incomplete. We do not know the exact reasons for the missing data in each variable and record; however, the CONVINCE trial was pragmatic, and all data were collected as part of routine clinical care, which resulted in missing information for some clinical and laboratory variables. Answering the questionnaire and participation in the physical performance test was voluntary, which also contributed to incomplete datasets. When comparing complete cases with those that have any missing data, it is noticeable that individuals without missing data are somewhat overrepresented in Eastern Europe and underrepresented in Western and Southern Europe. Apart from this regional difference, there are only negligible differences in other variables between the two groups.

To address missing values, we initially employed multiple imputation using the R package ‘mice’ version 3.16.0 to generate five imputed datasets (Van Buuren and Groothuis-Oudshoorn, 2011). Missing at random was assumed. We used the default settings of the mice package, i.e., predictive mean matching was used for numeric data, logistic regression imputation for binary data, polytomous regression imputation for categorical data, and a proportional odds model for ordinal data (Van Buuren and Groothuis-Oudshoorn, 2011). Incomplete variables were imputed under fully conditional specification, meaning that each variable was imputed conditionally on the others in an iterative process. All variables used in the regression analyses were included in the imputation process, except for one derived outcome variable (the difference between performance-based and patient-reported physical function), which was calculated after data imputation.

As a sensitivity analysis, we compared the resulting sample characteristics with those obtained from using another multiple imputation method (number of imputations = 5), namely classification and regression trees (CART; see Van Buuren and Groothuis-Oudshoorn, 2011), and those obtained from a complete case analysis (see Appendix Table A2). The sample characteristics demonstrated a high degree of similarity across the imputed datasets, both within each imputation method and between the different imputation methods. For example, the Pearson correlation between the first imputed datasets from the standard imputation method used by the mice package and the first imputed datasets from the CART method for the variables with the highest amount of imputed values (i.e., serum creatinine, performance-based physical function, symptom burden, and SCI) ranged between 0.92 and 0.99. Moreover, both imputation methods led to high similarities in sample characteristics with those resulting from complete case analysis (see Appendix Table A2), which supports the plausibility of the missing at random assumption.

Since this suggested a negligible impact resulting from the use of a specific imputed dataset, and since only a few variables were affected by more than 10% missing data, we decided to use only the first imputed dataset resulting from the standard method for further analysis. This is in line with the recommendation of Van Buuren (2018) not to use multiple imputation for further analysis if simpler methods are valid (Chapter 12.1.3).

Finally, restricting the regression analyses to the complete cases led to similar conclusions as the regression results based on the imputed dataset. This suggests robustness in the findings across different methods of handling missing data and supports the validity of the chosen approach in capturing the relationships among variables.

**Reference:**

Van Buuren, S. (2018). Flexible imputation of missing data: CRC press.

Van Buuren, S., & Groothuis-Oudshoorn, K. (2011). mice: Multivariate imputation by chained equations in R. Journal of Statistical Software, 45, 1-67.
