# Supplementary material for: Using a measurement type-independent metric to compare patterns of determinants between patient-reported versus performance-based physical function in hemodialysis patients
Source: Qual Life Res. 2024 Aug 5;33(11):2987–3001. doi: 10.1007/s11136-024-03745-6 (PMC11541257; doi:10.1007/s11136-024-03745-6)
Supplement: Supplementary file 4 — Supplementary file4 (DOCX 51 KB) [file 11136_2024_3745_MOESM4_ESM.docx]

| Table A4: Sensitivity analysis of the regression models with performance-based physical function as indicated by the PPT as outcome – Comparison of regression coefficients between different methods | | | | | | | |
| --- | --- | --- | --- | --- | --- | --- | --- |
| **Determinants** | **Model 1 (including SCI)** | | | | **Model 2 (with individual SCI components)** | | |
|  | **Beta (stepwise)** | | **95% CI** | **Beta (LASSO)** | **Beta (stepwise)** | **95% CI** | **Beta (LASSO)** |
| SCI | 0.73 | | 0.53; 0.92 | 0.74 |  |  |  |
| Age |  | |  |  | -0.24 | -0.27; -0.20 | -0.23 |
| Sex |  | |  |  |  |  | 0.55 |
| BMI | -0.21 | | -0.29; -0.11 | -0.20 | -0.20 | -0.29; -0.12 | -0.20 |
| Educational level† |  | |  |  |  |  |  |
| Lower secondary | 2.16 | | 0.83; 3.49 | 2.01 | 1.61 | 0.41; 2.91 | 1.54 |
| Upper secondary | 4.35 | | 2.98; 5.67 | 4.19 | 2.63 | 2.35; 4.90 | 3.63 |
| Tertiary | 2.81 | | 1.09; 4.44 | 2.70 | 2.26 | 0.65; 3.85 | 2.32 |
| Laboratory measures |  | |  |  |  |  |  |
| Hemoglobin | 0.28 | | -0.11; 0.68 | 0.33 | 0.36 | -0.03; 0.75 | 0.40 |
| Calcium |  | |  | -0.19 |  |  | -0.11 |
| Phosphate |  | |  | -0.09 |  |  | -0.25 |
| Serum creatinine |  | |  |  | -2.22 | -0.55; 0.11 | 0.13 |
| Sodium | 0.20 | | 0.06; 0.35 | 0.19 | 0.23 | 0.10; 0.36 | 0.22 |
| Kt/V |  | |  |  | -1.47 | -2.53; -0.51 | -1.46 |
| Comorbidities |  | |  |  |  |  |  |
| Diabetes | -2.32 | -3.43; -1.36 | | -2.35 | -2.13 | -3.04; -1.13 | -2.06 |
| CVD | -2.84 | | -3.76; -1.85 | -2.82 | -1.72 | -2.70; -0.82 | -1.60 |
| Cancer |  | |  | 0.57 | 1.70 | 0.49; 2.88 | 1.73 |
| COPD | -2.27 | | -4.11; -0.54 | -2.23 | -1.44 | -3.15; 0.42 | -1.35 |
| Treatment-related factors |  | |  |  |  |  |  |
| Dialysis vintage |  | |  | -0.02 |  |  | -0.02 |
| ESA |  | |  | 0.27 |  |  | 0.32 |
| Iron preparations | 2.61 | | 1.61; 3.53 | 2.53 | 2.50 | 1.53; 3.39 | 2.42 |
| Patient-reported variables |  | |  |  |  |  |  |
| Depression | -0.08 | | -0.17; 0.00 | -0.07 | -0.11 | -0.20; -0.03 | -0.10 |
| Fatigue | -0.07 | | -0.14; 0.00 | -0.06 | -0.06 | -0.13; 0.00 | -0.05 |
| Anxiety | 0.07 | | 0.00; 0.15 | 0.06 | 0.06 | -0.01; 0.14 | 0.06 |
| Sleep disturbance |  | |  | 0.03 |  |  | 0.03 |
| Pain interference | -0.13 | | -0.19; -0.06 | -0.12 | -0.12 | -0.18; -0.06 | -0.11 |
| Symptom burden |  | |  | -0.04 |  |  | -0.05 |
| Self-efficacy | 0.15 | | 0.00; 0.29 | 0.16 | 0.12 | -0.02; 0.26 | 0.13 |
| Perceived Stress |  | |  | 0.05 |  |  | 0.06 |
| Region^§^ |  | |  |  |  |  |  |
| Eastern European | -2.22 | | -3.35; -0.95 | -2.08 | -2.84 | -4.02; -1.72 | -2.83 |
| Southern European | -1.50 | | -2.60; -0.35 | -1.47 | -1.92 | -3.02; -0.87 | -1.85 |
| *Abbreviations:* BMI, body mass index; CCA, complete case analysis; CI, confidence interval; COPD; chronic obstructive pulmonary disease; CVD, cardiovascular disease; ESA, erythropoiesis-stimulating agent; Kt/V, a measure of hemodialysis adequacy; LASSO, Least Absolute Shrinkage and Selection Operator; *n.a.*, not applicable; PPT, Physical Performance Test; SCI, simplified creatinine index.  † Reference category: “Less than secondary”  § Reference category: “Western European” | | | | | | | |

| Table A5: Sensitivity analysis of the regression models with patient-reported physical function as indicated by the PROMIS-PF4a as outcome – Comparison of regression coefficients between different methods | | | | | | |
| --- | --- | --- | --- | --- | --- | --- |
| **Determinants** | **Model 1 (including SCI)** | | | **Model 2 (with individual SCI components)** | | |
|  | **Beta (stepwise)** | **95% CI** | **Beta (LASSO)** | **Beta (stepwise)** | **95% CI** | **Beta (LASSO)** |
| SCI | 0.54 | 0.38; 0.69 | 0.53 |  |  |  |
| Age |  |  |  | -0.14 | -0.17; -0.11* | -0.13 |
| Sex |  |  |  | -0.98 | -1.66; -0.22 | -0.76 |
| BMI | -1.12 | -0.18; -0.06 | -0.12 | -0.11 | -0.17; -0.04 | -0.09 |
| Educational level† |  |  |  |  |  |  |
| Lower secondary | 1.20 | 0.11; 2.25 | 0.81 | 0.92 | -0.12; 2.03 |  |
| Upper secondary | 2.03 | 1.00; 3.16 | 1.60 | 1.60 | 0.50; 2.68 | 0.64 |
| Tertiary | 1.49 | 0.31; 2.77 | 1.05 | 1.16 | -0.04; 2.31 | 0.09 |
| Laboratory measures |  |  |  |  |  |  |
| Hemoglobin | 0.38 | 0.09; 0.63 | 0.34 | 0.41 | 0.15; 0.67 | 0.31 |
| Calcium |  |  | -0.21 |  |  |  |
| Phosphate |  |  | 0.02 |  |  |  |
| Serum creatinine |  |  |  |  |  | 0.06 |
| Sodium | 0.08 | -0.02; 0.20 | 0.07 | 0.10 | 0.00; 0.20 | 0.06 |
| Kt/V |  |  |  | -0.59 | -1.34; 0.09 | -0.40 |
| Comorbidities |  |  |  |  |  |  |
| Diabetes | -1.56 | -2.40; -0.76 | -1.58 | -1.59 | -2.37; -0.86 | -1.45 |
| CVD | -1.91 | -2.65; -1.18 | -1.87 | -1.39 | -2.20; -0.62 | -1.22 |
| Cancer | -0.84 | -1.78; 0.14 | -0.62 |  |  |  |
| COPD | -2.46 | -3.71; -1.10 | -2.36 | -2.21 | -3.45; -1.06 | -1.79 |
| Treatment-related factors |  |  |  |  |  |  |
| Dialysis vintage |  |  | -0.02 |  |  |  |
| ESA |  |  | -0.24 |  |  |  |
| Iron preparations |  |  | 0.31 |  |  |  |
| Patient-reported variables |  |  |  |  |  |  |
| Depression | -0.07 | -0.12; -0.01 | -0.08 | -0.08 | -0.14; -0.03 | -0.08 |
| Fatigue | -0.24 | -0.29; -0.18 | -0.23 | -0.23 | -0.28; -0.17 | -0.23 |
| Anxiety |  |  | 0.02 |  |  |  |
| Sleep disturbance |  |  |  |  |  |  |
| Pain interference | -0.21 | -0.26; -0.16 | -0.21 | -0.20 | -0.25; -0.15 | -0.20 |
| Symptom burden | -0.12 | -0.17; -0.07 | -0.13 | -0.12 | -0.17; -0.07 | -0.12 |
| Self-efficacy | 0.18 | 0.06; 0.30 | 0.18 | 0.17 | 0.06; 0.30 | 0.16 |
| Perceived Stress | -0.07 | -0.18; 0.03 | -0.06 |  |  | -0.03 |
| Region^§^ |  |  |  |  |  |  |
| Eastern European |  |  | 0.12 |  |  |  |
| Southern European |  |  |  |  |  |  |
| *Abbreviations:* BMI, body mass index; CCA, complete case analysis; CI, confidence interval; COPD; chronic obstructive pulmonary disease; CVD, cardiovascular disease; ESA, erythropoiesis-stimulating agent; Kt/V, a measure of hemodialysis adequacy; LASSO, Least Absolute Shrinkage and Selection Operator; *n.a.*, not applicable; PROMIS-PF4a, Patient-Reported Outcomes Measurement Information System Physical Function 4a; SCI, simplified creatinine index.  † Reference category: “Less than secondary”  § Reference category: “Western European” | | | | | | |

| Table A6: Sensitivity analysis of the regression models with T-score differences between PROMIS-PF4a and PPT as dependent variable as outcome – Comparison of regression coefficients between different methods | | | | | | |
| --- | --- | --- | --- | --- | --- | --- |
| **Determinants** | **Model 1 (including SCI)** | | | **Model 2 (with individual SCI components)** | | |
|  | **Beta (stepwise)** | **95% CI** | **Beta (LASSO)** | **Beta (stepwise)** | **95% CI** | **Beta (LASSO)** |
| SCI | -0.19 | -0.40; -0.01 | -0.16 |  |  |  |
| Age |  |  |  | 0.11 | 0.08; 0.14 | 0.09 |
| Sex |  |  |  | -1.42 | -2.34; -0.53 | -1.11 |
| BMI | 0.08 | 0.00; 0.17 | 0.06 | 0.10 | 0.03; 0.18 | 0.07 |
| Educational level† |  |  |  |  |  |  |
| Lower secondary | -0.97 | -2.27; 0.24 | -0.05 |  |  |  |
| Upper secondary | -2.37 | -3.68; -1.00 | -1.30 | -1.36 | -2.26; -0.42 | -1.20 |
| Tertiary | -1.34 | -2.95; 0.16 | -0.33 |  |  | -0.33 |
| Laboratory measures |  |  |  |  |  |  |
| Hemoglobin |  |  |  |  |  |  |
| Calcium |  |  |  |  |  |  |
| Phosphate |  |  | 0.03 | 0.24 | -0.06; 0.54 | 0.13 |
| Serum creatinine |  |  |  |  |  |  |
| Sodium | -0.12 | -0.25; 0.02 | -0.08 | -0.15 | -0.27; -0.03 | -0.10 |
| Kt/V |  |  |  | 0.93 | 0.02; 1.95 | 0.65 |
| Comorbidities |  |  |  |  |  |  |
| Diabetes | 0.78 | -0.11; 1.70 | 0.72 |  |  | 0.47 |
| CVD | 0.89 | 0.00; 1.73 | 0.71 |  |  | 0.03 |
| Cancer | -1.36 | -2.51; -0.09 | -1.07 | -1.98 | -3.20; -0.80 | -1.63 |
| COPD |  |  |  |  |  | -0.26 |
| Treatment-related factors |  |  |  |  |  |  |
| Dialysis vintage |  |  | 0.00 |  |  |  |
| ESA | -0.71 | -1.69; 0.26 | -0.55 |  |  | -0.47 |
| Iron preparations | -2.09 | -3.15; -1.23 | -1.88 | -2.13 | -2.97; -1.27 | -1.83 |
| Patient-reported variables |  |  |  |  |  |  |
| Depression |  |  | -0.01 |  |  | 0.00 |
| Fatigue | -0.18 | -0.25; -0.12 | -0.17 | -0.18 | -0.25; -0.12 | -0.18 |
| Anxiety | -0.05 | -0.10; 0.01 | -0.03 |  |  | -0.02 |
| Sleep disturbance |  |  | 0.03 | -0.04 | -0.09; 0.02 | -0.03 |
| Pain interference | -0.09 | -0.15; -0.04 | -0.08 | -0.09 | -0.15; -0.04 | -0.08 |
| Symptom burden | -0.10 | -0.17; -0.02 | -0.09 | -0.09 | -0.17; -0.02 | -0.08 |
| Self-efficacy |  |  |  |  |  | 0.01 |
| Perceived Stress | -0.13 | -0.25; -0.01 | -0.08 | -0.13 | -0.24; -0.01 | -0.09 |
| Region^§^ |  |  |  |  |  |  |
| Eastern European | 2.41 | 1.27; 3.55 | 1.77 | 2.72 | 1.59; 3.72 | 2.16 |
| Southern European | 1.58 | 0.58; 2.51 | 1.27 | 1.87 | 0.83; 2.83 | 1.36 |
| *Abbreviations:* BMI, body mass index; CCA, complete case analysis; CI, confidence interval; COPD; chronic obstructive pulmonary disease; CVD, cardiovascular disease; ESA, erythropoiesis-stimulating agent; Kt/V, a measure of hemodialysis adequacy; LASSO, Least Absolute Shrinkage and Selection Operator; *n.a.*, not applicable; PPT, Physical Performance Test; PROMIS-PF4a, Patient-Reported Outcomes Measurement Information System Physical Function 4a; SCI, simplified creatinine index.  † Reference category: “Less than secondary”  § Reference category: “Western European” | | | | | | |
